# A novel dynamic Optuna hybrid Harris Hawks Optimization approach for classification of CAD

**DOI:** 10.1371/journal.pone.0353605

**Published:** 2026-07-17

**Authors:** C. K. Revathi, H. Santhi

**Affiliations:** 1 Full-time research scholar, School of Computer Science and Engineering, Vellore Institute of Technology, Vellore, Tamil Nadu, India; 2 Associate Professor Senior, School of Computer Science and Engineering, Faculty of Database Management Systems, Vellore Institute of Technology, Vellore, Tamil Nadu, India; King Abdulaziz University, SAUDI ARABIA

## Abstract

Coronary Artery Disease (CAD) is a leading cause of mortality worldwide and is primarily associated with atherosclerotic plaque formation, resulting in coronary artery stenosis. The accurate prediction of CAD using deep learning models is often constrained by the limitations of conventional optimization techniques, including premature convergence and limited adaptability of the models. To address these challenges, this study proposes a Dynamic Optuna Hybrid Harris Hawks Optimization (Dynamic Optuna H-HHO) framework to enhance the performance of deep learning–based CAD prediction models. The proposed approach integrates dynamic parameter adjustment, adaptive escape energy mechanisms, and Optuna-based hyperparameter tuning. The framework was applied to optimize several deep-learning classifiers, including ResNet-50, VGG-16, InceptionV3, and MobileNet, using a coronary artery stenosis dataset. The performance was evaluated through a comparative analysis with models optimized using the conventional Hybrid Harris Hawks Optimization (H-HHO) algorithm. The experimental results indicate that the proposed Dynamic Optuna H-HHO framework consistently improves the predictive accuracy across all evaluated models. InceptionV3 achieved the highest accuracy of 97.9%, followed by MobileNet with 97.6%, compared with the maximum accuracy of 82.46% obtained using traditional HHO-based optimization. By combining adaptive optimization strategies with automated hyperparameter tuning, the proposed framework provides a robust and scalable solution for improving the accuracy of coronary artery disease prediction.

## 1. Introduction

Cardiovascular disease (CVD) represents a broad spectrum of disorders of the heart and vascular system continues to be a major global health challenge. According to large-scale epidemiological studies, CVD causes almost 1/3 of all deaths worldwide. Pneumonia is the leading cause of death worldwide [1]. Coronary artery disease (CAD), also referred to as ischemic heart disease, is the most prevalent and life-threatening form of cardiovascular disease, affecting both men and women of all age groups [1,2]. The global burden of CAD alone is involved in millions of deaths each year, and reports assessments, in particular, the urgent need to realize accurate risk prediction and early diagnosis frameworks [1].

The early identification of CAD risk factors enables timely clinical intervention and significantly improves patient outcomes. Predictive modeling plays an important role not only in early diagnosis. and for designing personalized preventive and therapeutic strategies. Several modifiable and non-modifiable risk factors are associated with CAD, including tobacco use, physical inactivity, hypertension, obesity, dyslipidemia, diabetes mellitus, and unhealthy dietary habits [3]. These risk factors frequently interact synergistically, amplifying disease progression owing to the complex multifactorial pathophysiology of CAD.

In recent years, deep learning (DL) has made its mark in biomedical image analysis thanks to its powerful, superior capability to learn hierarchical and discriminative features from complex medical datasets [4–6]. DL-based models have demonstrated remarkable performance in automated coronary artery segmentation, stenosis detection, and disease classification from X-ray coronary angiography images. Such Advancements have addressed the long-standing challenges of diagnostic accuracy, interobserver variability, and clinical workload in cardiovascular imaging [7–10].

Optimization, which involves the systematic tuning of DL models, plays a pivotal role in strengthening the performance of DL models. Model parameters and hyperparameters are used to achieve objectives such as improved accuracy, reduced error, and faster convergence [11,12]. Optimization techniques are employed throughout the model development lifecycle, including training, [7–10] validation, and deployment, and are essential for robust and consistent generalizable performances. In a broader sense, optimization refers to selecting the best possible solution from a set of alternatives that maximizes efficiency and effectiveness, a concept widely applied in mathematics, engineering, and machine learning [11]. The growing complexity of modern computational problems has driven increased interest in advanced and nature-inspired optimization strategies, particularly in cases where conventional methods fail to scale effectively [12].

Harris hawk optimization (HHO) is a population-based metaheuristic inspired by the cooperative hunting behavior of Harris hawks. The algorithm operates in two principal phases: exploration, which encourages a global search in the solution space, and exploitation, which focuses on refining candidate solutions using energy-based strategies that mimic prey pursuit and surprise pounce mechanisms [13–16]. The transition between these phases is governed by a dynamic energy model that allows a balanced trade-off between diversification and intensification during optimization. HHO has gained significant attention because of its high global search capability and adaptability in medical and healthcare applications. It has been successfully applied to the diagnosis of heart diseases. using artificial neural networks [17,18], hybrid adaptive and genetic learning frameworks for cardiovascular classification [19–21], and CAD prediction using support vector machines optimized by HHO [22]. Context-aware hybrid HHO models have demonstrated improved performance in CAD risk prediction tasks [23]. These studies collectively highlight the suitability of HHO for the formation of complex diagnostic problems in healthcare.

Beyond healthcare, HHO and its variants have demonstrated effectiveness in diverse optimization problems, including hybrid DE–HHO frameworks for forecasting and regression tasks, [24] predictive modeling using k-fold cross-validation, [25] and enhanced global optimization through improved exploration–exploitation mechanisms [13–16]. These applications confirm HHO’s robustness and flexibility in a wide range of optimization domains. Despite significant progress in DL-based medical image analysis, particularly for CAD detection from angiographic images, model performance remains highly sensitive to hyperparameter selection. Inadequate tuning can lead to underfitting, overfitting, or slow convergence, thereby reducing the diagnostic reliability. Classic tuning techniques, such as grid and random searches, are computationally expensive and inefficient for high-dimensional datasets.

To address these challenges, this study proposes a Dynamic Optuna Hybrid Harris Hawks Optimization (Dynamic Optuna H-HHO) framework. The proposed approach integrates the global exploration strength of HHO with Optuna’s adaptive and intelligent hyperparameter optimization mechanism, enabling the automated, efficient, and dynamic tuning of deep learning models for CAD classification. By synergistically combining Biologically inspired optimization with modern hyperparameter search strategies, the proposed method will develop a robust, scalable, and clinically viable diagnostic framework for real-world coronary artery disease applications, supported by recent angiographic datasets and benchmarking studies [26–30].

### 1.1. Related work

Harris Hawks Optimization (HHO) has garnered significant research interest as a nature-inspired metaheuristic owing to its effective balance between exploration and exploitation, guided by an energy-based hunting strategy. In our early efforts, we concentrated on the core structure of the HHO algorithm to improve convergence stability and global search capability. Zhang and colleagues [13] introduce a modified HHO framework to improve convergence behavior in high-dimensional optimization problems.

Qu et al. [14] incorporated information-exchange mechanisms among hawks to preserve population diversity and ameliorate premature convergence. Sihwail et al [15] further increased HHO by: integration of feature selection strategies. Fan et al. [16] proposed a quasi-reflected HHO variant to strengthen exploration efficiency and accelerate convergence. Based on these algorithmic enhancements, HHO has been widely adopted in healthcare applications, particularly for disease diagnosis and risk-prediction. Al-Safi (et al. [17]) used HHO together with artificial neural networks (ANNs) for heart disease diagnosis, reporting improved classification accuracy. Alsafi et al. [18] extended the idea to the diagnosis of general disease with HHO-based ANN frameworks. Balamurugan and colleagues [19] proposed an adaptive HHO integrated

The enhanced genetic algorithm for heart disease classification achieved notable performance improvements. Kumar and Rekha [20] introduced an improved HHO-based learning framework for cardiovascular disease prediction and demonstrated enhanced diagnostic reliability. Prasanna and Challa [21] combined binary HHO with a deep Bi-LSTM architecture for heart risk prediction using HHO (Another Application of HHO in Medical Time Series Data). Maleki et al. [22] Optimized support vector machines based on HHO for coronary artery disease prediction, while Vijayaraj and Pasupathi [23] proposed a context-aware hybrid HHO framework for personalized CAD risk assessment. Beyond medical diagnosis, HHO has been successfully applied to complex engineering optimization tasks. A hybrid differential evolution–Harris hawks optimization (DE–HHO) algorithm was proposed by Fu et al. [24] for optimizing kernel extreme learning machines, achieving superior accuracy and computational efficiency in wind speed forecasting. Moayedi et al. [25] integrated HHO with k-fold cross-validation for slope stability prediction, demonstrating reliable modeling of nonlinear relationships in real-world datasets. Despite these advancements, a common limitation of most existing HHO-based and hybrid approaches is their reliance on static manually tuned control parameters. Fixed exploration coefficients, escape energy settings, and learning-related parameters restrict the ability of the optimizer to adapt to dynamically changing the search landscape. This limitation becomes particularly pronounced when (HHO) is applied to high-dimensional and complex problems (e.g., * -) in deep learning–based coronary artery disease classification, where inappropriate parameter settings can lead to premature convergence, inefficient exploration, or unstable training behavior.

Motivated by these gaps, the proposed Dynamic Optuna Hybrid Harris Hawks Optimization (Dynamic Optuna H-HHO) framework directly extends prior HHO-based research by introducing adaptive and automated parameter tuning into the optimization process. By integrating Optuna’s dynamic hyperparameter sampling strategy with HHO’s biologically inspired hunting mechanism, the proposed method enables the real-time adjustment of critical parameters, such as escape energy, exploration–exploitation coefficients, and learning rates. Unlike existing orthologs and hybrids of HHO [13–25], which use fixed or heuristically correct defined configurations, the Dynamic Optuna H-HHO framework continuously adapts to the evolving search landscape. This design choice provides improved convergence stability, enhanced global search efficiency, and superior generalization performance, making it particularly suitable for deep-learning-based CAD diagnosis in real-world clinical settings.

These studies highlight the versatility of HHO; however, a key limitation remains in that most existing implementations employ static configurations, lacking dynamic adaptation to the evolving search landscape or learning objectives.

However, HHO faces inherent limitations,

- The algorithm spends significant time in the exploration phase in early iterations, which slows convergence, and the exploitation phase lacks a mechanism to refine the solution, which can lead to suboptimal convergence and stagnation of the algorithm.
- The static nature of the algorithm renders it less suitable for real-time and adaptive scenarios.
- The escaping Energy(E) and movement strategies were static and predefined. This creates optimization problems, and the algorithm is less flexible.
- Poor initialization can lead to slower convergence or an optimal solution.

To address this limitation, the proposed work will explore several improvement strategies, which are briefly described below.

1. We proposed a novel Dynamic Optuna Hybrid Harris Hawks Optimization (Dynamic Optuna H-HHO) algorithm that introduced dynamic adjustment of step size, velocity, and escaping energy, thereby addressing the stagnation and inflexibility of traditional HHO and other metaheuristics.
2. We integrated the proposed optimization framework with four state-of-the-art deep learning classifiers such as ResNet50, VGG16, InceptionV3, and MobileNet. We demonstrated its effectiveness in enhancing the classification performance on a real-world coronary stenosis image dataset.
3. We empirically demonstrated that Optuna H-HHO significantly outperformed both conventional HHO and standard classifiers, achieving up to 97.9% accuracy and showing improved F1 scores and AUC across all the tested models.
4. We compared our proposed approach with traditional optimizers such as Genetic Algorithm, PSO, GWO and demonstrated how the proposed method overcame key limitations, such as static parameters and premature convergence, through dynamic tuning and adaptive control.
5. We highlighted the clinical utility and deployability of the framework, emphasizing its ability to support real-time CAD diagnosis in clinical decision support systems through high accuracy, lightweight model compatibility, and future integration with interpretability tools, such as Grad-CAM, LIME, and SHAP.

Hence, this study proposed a novel hybrid algorithm called Dynamic Optuna H-HHO, which merged Optuna with H-HHO to overcome the limitations of H-HHO. This amalgamated approach integrated three unique strategies to augment its overall effectiveness. The main contributions of this study are as follows.

- Primarily, dynamically adjusting parameters, such as the step size (α) or energy (E), enables the algorithm to adjust progressively to the optimization landscape. Early iterations focused on exploration (broad search), whereas later iterations prioritized exploitation (fine–tuning).
- Secondly, the best solution found so far (Xrab) should be retained and not replaced by inferior solutions, as this helps preserve high-quality results and supports stable convergence.
- Finally, adjusting the exploration and exploitation phases based on fitness improves efficiency by balancing global search to avoid local optima and local refinement for faster and more accurate optimization..

The primary novelty of this work lies in the development of a dynamic hybrid hyperparameter optimization framework that integrates Optuna with Harris Hawks Optimization. Unlike conventional tuning approaches that rely on either probabilistic search or population-based optimization independently, the proposed Dynamic Optuna–H-HHO framework combines adaptive trial-based parameter sampling with the exploration–exploitation capability of HHO to improve optimization efficiency in high-dimensional hyperparameter spaces. The proposed strategy dynamically regulates the search process to enhance convergence stability and reduce the likelihood of premature convergence. Furthermore, the framework is applied to optimize deep learning models for coronary artery disease classification using multiple CNN architectures, including ResNet50, VGG16, InceptionV3, and MobileNet. The effectiveness of the proposed framework is further validated through rigorous statistical analysis using the Friedman Test and Wilcoxon Signed-Rank Test, demonstrating its superiority over the baseline optimization approach.

**Fig 1:**
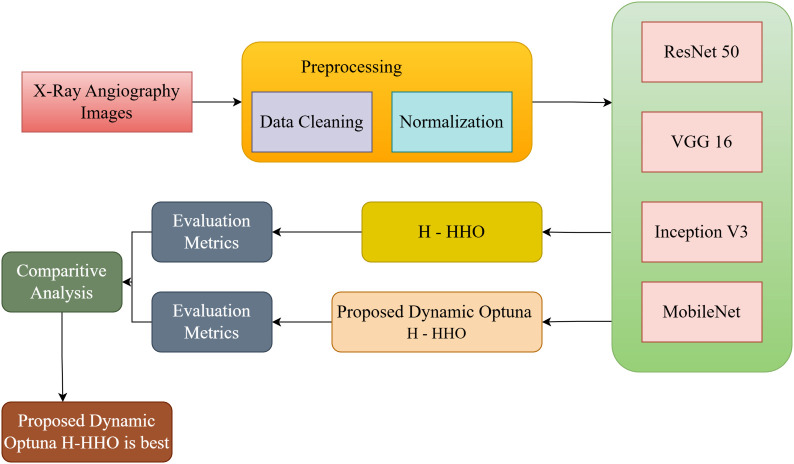
Architecture diagram of the Proposed Dynamic Optuna H-HHO.

We conducted extensive experiments to assess the efficacy and robustness of the Dynamic Optuna H-HHO algorithm, employing various classifiers such as ResNet50, VGG16, InceptionV3, and MobileNet. Initially, experimental testing was performed using the stenosis dataset. We employed Dynamic Optuna H-HHO to optimize the classifier parameters, leading to notable enhancements in the overall performance and effectiveness of the model. Our research focuses on using Dynamic Optuna H-HHO to enhance CAD prediction by optimizing classifier parameters and presenting a novel hybrid model that merges features from both Optuna and H-HHO. Fig 1 illustrates the architecture of our proposed method.

This paper is organized as follows. Section 2 presents a detailed description of the proposed model and its optimization strategy. Section 3 reports the numerical results and compares the proposed approach with existing methods. Finally, Section 4 summarizes the main findings and outlines directions for future research.

## 2. Methodology

### 2.1. Data acquisition

This study used a publicly available angiographic dataset comprising coronary angiography image series from 100 patients with clinically confirmed one-vessel coronary artery disease The dataset included X-ray angiographic images from 100 individuals who underwent coronary angiography. The dataset consisted of 8,325 grayscale images with dimensions ranging from 512 × 512–1000 × 1000 pixels. These images were captured using two systems: Siemens Coroscop and GE Healthcare Innova. The dataset is publicly available at Mendeley Data (https://doi.org/10.17632/ydrm75xywg.1). This dataset was previously employed in the research titled “Real-time Coronary Artery Stenosis Detection Based on Modern Neural Networks” and now underpins ongoing studies on advanced image analysis methods for detecting coronary artery disease. The dataset was allocated with 70% for 15% for training, 15% for validation and the remaining 15% for testing; all experiments were conducted on a system with an NVIDIA

GPU (16 GB VRAM), Intel i7 CPU, and 32 GB RAM implementation used Python 3.9, TensorFlow/Keras, Optuna, NumPy, OpenCV, and Scikit-learn. The average training time ranged between 2 and 4 h per model, depending on the architecture and optimization iterations.

### 2.2. Data preprocessing

Preprocessing involved three essential operations: color space conversion, resizing, and normalization. The preprocessing began with converting all grayscale images to RGB format to achieve uniform input channels, which matched the requirements of the CNNs. All images were resized to a uniform size of 224 × 224 pixels, maintaining the same spatial size for all images in the dataset. The values of the pixel intensities were normalized from the original scale [0, 255] to [0, 1]. Normalization facilitates the stability and efficiency of training by keeping the input values within a more standardized range, which supports better optimization during both training and inference of the model. Explicit vessel segmentation and aggressive data augmentation were intentionally not applied to preserve anatomical integrity and to align with established angiography-based CAD

### 2.3. Architecture of baseline classifiers

In this study, we employed four foundational classifiers to categorize coronary artery disease: ResNet-50, VGG-16, Inception-V3, and Mobile-Net. The primary objective of this study is to evaluate the effectiveness and generalizability of the proposed Dynamic Optuna H-HHO optimization framework across diverse deep learning models. Therefore, four well-established convolutional neural network architectures—ResNet-50, VGG-16, Inception-V3, and MobileNet—were selected as baseline classifiers.

#### 2.3.1. ResNet50.

Recent studies have demonstrated the effectiveness of deep and transfer learning approaches for coronary artery disease (CAD) analysis using X-ray angiographic images. Deep convolutional networks have shown strong capability in learning discriminative features for automated coronary stenosis detection [31], whereas transfer learning reduces computational cost and improves performance in scenarios with limited annotated medical data [32]. Prior research has further emphasized that careful model selection and systematic optimization can surpass several existing state-of-the-art stenosis detection methods [33]. Additionally, ResNet-50 has demonstrated strong generalization across multiple medical imaging domains through successful fine-tuning in chest X-ray–based disease classification tasks [34].

Motivated by these findings, ResNet-50 was adopted in this study as a pre-trained deep feature extractor and baseline classifier for CAD detection using X-ray angiographic images. In the proposed framework, each angiographic image is first preprocessed and then passed through ResNet-50, where initial convolution and pooling layers extract low-level vascular features such as edges and contrast gradients. The image then propagates through stacked residual bottleneck blocks, which learn hierarchical representations of vessel continuity and stenotic narrowing using residual mappings that ensure stable gradient flow and efficient deep training. After deep feature extraction, global average pooling compresses the learned feature maps into a compact representation, which is fed into a fully connected layer to generate CAD classification probabilities.

Importantly, ResNet-50 is not introduced as a novel architectural contribution; rather, it serves as a standardized and well-validated backbone model to objectively measure the dynamic impact of the proposed Optuna–Harris Hawks Optimization (H-HHO) strategy on convergence behavior, generalization capability, and overall classification performance. By keeping the feature extraction architecture fixed, the study isolates and evaluates the effectiveness of the optimization framework in enhancing training stability and predictive robustness.

#### 2.3.2. VGG16.

VGG-16 has been widely adopted in medical image analysis owing to its simple and uniform convolutional architecture, which enables effective hierarchical feature extraction from imaging data. Prior studies have demonstrated its utility in coronary artery disease analysis and vessel-related tasks, particularly when combined with transfer learning to mitigate limited dataset sizes and computational constraints [26–28,31,32,35].

In this work, VGG-16 is applied to X-ray angiographic images to capture spatial patterns related to coronary vessel morphology and stenotic regions through stacked 3 × 3 convolutional layers and progressive max-pooling operations. As the network deepens, it learns increasingly abstract representations of vascular continuity and narrowing patterns relevant to CAD detection, with final classification performed via fully connected layers and softmax activation.

Importantly, VGG-16 is selected as a classical deep architecture without residual or multi-scale connections, enabling direct comparison with more advanced models and allowing systematic assessment of how architectural depth and dynamic optimization influence CAD classification performance. While the feature extraction structure remains unchanged, the Dynamic Optuna–H-HHO strategy adaptively tunes key hyperparameters governing the training process, ensuring fair evaluation of the optimization framework’s impact on convergence stability, generalization, and predictive robustness.

#### 2.3.3. Inceptionv3.

Inception-V3 incorporates multi-scale feature extraction through parallel convolutional paths (the network must capture both local information and worldwide contextual information within an image). This architectural property is particularly relevant for coronary angiographic analysis, where stenotic regions exhibit substantial variations in size, shape, and visual appearance. Previous studies [27,29] have shown that multiscale representations can enhance performance in coronary stenosis detection and angiographic image analysis.

InceptionV3 processes CAD X-ray angiographic images using a multi-scale convolutional architecture in which parallel convolutional filters (e.g., 1 × 1 and 3 × 3) operate within Inception modules to capture vessel features at different spatial resolutions. After initial convolution and pooling layers extract low-level vascular patterns such as edges and contrast gradients, the stacked Inception blocks learn higher-level representations of vessel morphology and stenotic narrowing. Global average pooling and a final softmax layer then produce CAD classification probabilities.

In the proposed framework, Inception-V3 is used as a baseline deep learning model for evaluating the impact of the Dynamic Optuna H-HHO optimization strategy on architectures with inherent multiscale representation capability. The network was fine-tuned on angiographic images under strictly controlled patient-level data splitting to prevent data leakage. By including Inception-V3, this study enables a systematic assessment of how advanced architectural design, when combined with metaheuristic-driven hyperparameter optimization, influences the classification accuracy and generalization performance.

#### 2.3.4. Mobilenet.

MobileNet is a lightweight deep-learning architecture designed to achieve a favorable balance between computational efficiency and classification performance through depthwise separable convolutions. Recent studies have highlighted its suitability for medical imaging tasks, including coronary artery segmentation and disease detection, particularly in resource-constrained or real-time clinical environments [30].

MobileNet processes CAD X-ray angiographic images using depthwise separable convolutions, which reduce computational cost while preserving effective feature learning. After preprocessing, the network extracts low-level vascular features such as edges and contrast gradients, followed by inverted residual blocks that capture higher-level patterns including vessel continuity and stenotic narrowing. Global average pooling and a softmax layer then produce CAD classification probabilities.

MobileNet is included as a baseline lightweight model to evaluate whether the proposed Dynamic Optuna H-HHO framework can enhance performance without substantially increasing the computational cost. By optimizing MobileNet using the same experimental protocol as other architectures, this study assesses the adaptability and generalization of the optimization strategy across both heavy and lightweight networks. This comparison supports the practical relevance of the proposed approach for deployment-oriented CAD systems.

### 2.4. HHO(Harris Hawks Optimization) algorithm

The Harris Hawks’ collaborative hunting strategies form the basis of this meta-heuristic algorithm, which aims to optimize solutions through a balanced approach. Through exploration and exploitation, the method aims to achieve optimal results. Such a two-phase construction makes the algorithm capable of going through all those search stages and does not focus only on promising domains.

#### 2.4.1. Exploration phase.

In this phase, hunting behavior takes place. The HHO algorithm emulates exploratory action when navigating the search space by Eqn 1


$$\begin{array}{c} {\text{P}}^{\prime }(\text{t}+\text{1})=\;\left\{ \begin{array}{c} {\text{P}}_{\text{rand}}^{\prime }\;(\text{t})-{\text{r}}_{\text{1}}\;\left|\;{\text{P}}_{\text{rand}}^{\prime }\;(\text{t})-\text{2}{\text{r}}_{\text{2}}\;{\text{P}}^{\prime }\;(\text{t})\right|;\;\\ \left({\text{P}}_{\text{rabbit}}^{\prime }\;(\text{t})-{\text{P}}^{\prime }\text{ m}(\text{t})\right)-{\text{r}}_{\text{3}}\left(\text{LB}+\;{\text{r}}_{\text{4}}(\text{UB}-\text{LB})\right) \end{array}\;q<\text{0}.\text{5}\right. \end{array}$$ (1)

where

${\text{P}}^{\prime }(\text{t})$ – Hawk’s current position

$P_{rand}^{\prime }(t)$ Hawks randomly chose the present position.

$\left(r_{\text{1}}{,r}_{\text{2}},\;and\;q\right)$ The interval [0,1] was utilized for generating stochastic values.

The computation of the Average position for existing Hawks is facilitated by Eqn 2


$$\begin{array}{c} {\text{P}}_{\text{m}}^{\prime }(\text{t})=\;\frac{\text{1}}{\text{N}}\;\sum_{\text{i}=\text{1}}^{\text{N}}{\text{P}}_{\text{i}}^{\prime }\;(\text{t}) \end{array}$$ (2)

where

$P_{i}^{\prime }(t)$– spatial coordinates of hawks at each iteration ‘t’

N- total quantity of Hawks

#### 2.4.2. Progression between two phases Of H-HHO.

It transitions from a broad exploratory to a focused exploitation phase and is driven by an “Escape Energy,” represented by E.


$$\begin{array}{c} \text{E}=\text{2}{\text{E}}_{\text{0}}\left(\text{1}-\frac{\text{t}}{\text{T}}\right) \end{array}$$ (3)

where

$E_{\text{0}}$– initial energy cover from −1–1

t- current iteration

T – max iteration

#### 2.4.3. Exploitation phase.

In this phase, the chase is accurately emulated using four distinct strategies. One is a random number from 0 to 1, and Escaping energy E

Soft beige: When prey does not have the chance to escape, it makes some effort to escape from the place when |E| ≥ 0.5 and r ≥ 0.5. It is represented by Eqn 4


$$\begin{array}{c} P^{\prime }(t+\text{1})=\;\Delta \;P^{\prime }(t)-E|\;J\;P_{rabbit\;}^{\prime }(t)-\;P^{\prime }(t)| \end{array}$$ (4)

$$\begin{array}{c} \Delta P^{\prime }(t)=P_{rabbit}^{\prime }-P^{\prime }(t) \end{array}$$ (5)

where

$\Delta P^{\prime }(t)$– Difference between the current entity and the optimal entity of prey

J = 2 × (1 − r_5_) – Stochastic leap intensity of rabbit.

r_5_ – It ranges from 0 to 1

Hard Beige: When the prey is unable to escape due to insufficient escaping energy when |E| ≤ 0.5 and r ≥ 0.5

It is represented by Eq. 6


$$\begin{array}{c} P^{\prime }(t+\text{1})=\;P_{rabbit}^{\prime }(t)-E\;|\Delta \;P^{\prime }(t)| \end{array}$$ (6)

The fitness function of Soft beige and Hard beige is represented as


$$\begin{array}{c} P^{\prime }(t+\text{1})=\;\left\{ \begin{array}{c} Y^{\prime }\;;F\left(Y^{\prime }\right)<F\left(P^{\prime }\;(t)\right)\\ Z^{\prime }\;;F\left(Z^{\prime }\right)<F\left(P^{\prime }\;(t)\right) \end{array}\right. \end{array}$$ (7)

where

F- fitness value


$$\begin{array}{c} Y^{\prime }=\;P_{rabbit}^{\prime }(t)-E|\;JP_{rabbit\;}^{\prime }(t)-\;P_{m}^{\prime }(t)| \end{array}$$ (8)

$$\begin{array}{c} \text{Z}\text{'}=\;{\text{Y}}^{\prime }+\;{\text{s}}^{*}\text{ LF }(\text{D}) \end{array}$$ (9)

$$\begin{array}{c} \text{LF}(\text{x})=\text{0}.\text{01}*\;\frac{\text{u}*\;\sigma }{|\text{V}{|}^{\frac{\text{1}}{\beta }}}\;,\;\sigma {\left[\frac{\Gamma (\text{1}+\;\beta )*\text{sin}\left(\frac{\pi *\;\beta }{\text{2}}\right)}{\Gamma (\text{1}+\;\beta )*\beta *\text{2}\left(\frac{\beta -\text{1}}{\text{2}}\right)}\right]}^{\frac{\text{1}}{\beta }} \end{array}$$ (10)

Here, u and V are random numbers ranging from 0 to 1, and constant β is fixed at 1.5

### 2.5. Proposed dynamic optuna H-HHO algorithm

In our proposed system, the fundamental structure of a conventional HHO algorithm is incorporated with three key strategies: dynamic adjustments, preservation of the best solution, and an adaptive strategy.

#### 2.5.1. Exploration phase.

In the conventional HHO algorithm, this phase calculates the position using randomness in the global search using Eqs. (1) and (2), respectively. However, there was insufficient variability. Larger values enable faster movement, whereas smaller values refine precision. During immediate prey detection, randomness in the step length aids quick adjustments, whereas prolonged searches require a gradually decreasing step length for focused refinement. Early iterations emphasize broad exploration, whereas later iterations shift to detailed local search, balancing exploration and exploitation for improved efficiency and convergence. The modified exploration factor is modeled as follows in eqn 11


$$\begin{array}{c} ef=\;\left(b*rand-\;\frac{b}{\text{2}}\right)*\text{cos}\left(\frac{\pi }{\text{2}}\;,\;\frac{T_{max}-t}{T_{max}}\right) \end{array}$$ (11)

Here, b and rand are dynamically tuned by optuna after adjustment in the exploration phase $P^{\prime }(t+\text{1})$ is given by the eqn 12


$$\begin{array}{c} P^{\prime }(t+\text{1})=\left\{ \begin{array}{c} P_{rand}^{\prime }(t)-ef\;\left|\;P_{rand}^{\prime }(t)-\text{2}r_{\text{2}}P^{\prime }(t)\right|,\;q\;\geq \text{0}.\text{5}\\ \left(P_{rabbit}^{\prime }\;-\;P_{m}^{\prime }(t)\right)-ef\;\left(LB+\;r_{\text{4}}(UB-LB)\right)\;,\;q<\text{0}.\text{5} \end{array}\right. \end{array}$$ (12)

Velocity was introduced into the HHO algorithm, which dynamically guides population exploration, enhancing the capacity of the algorithm to effectively traverse the outcome domain. This modification addresses the challenge of the HHO becoming trapped in local optima by improving global exploration and balancing exploration and exploitation during the exploitation phase. It should be emphasized that this velocity term is not associated with any personal-best or global-best learning mechanisms. Instead, it serves as an auxiliary motion-control variable that regulates the step size and directional continuity of the original HHO energy-based update process. Equations 13 and 14 determine the velocity and position of an object in a soft beige.


$$\begin{array}{c} V_{i}(t+\text{1})=w\;V_{i}(t)+\;c_{\text{1}}*\;r_{\text{1}}\;\left(P_{best}\;(t)-P_{i}^{\prime }\;(t)\right)+\;c_{\text{2}}*r_{\text{2}}\left(G_{best}\;(t)-P_{i}^{\prime }\;(t)\right) \end{array}$$ (13)

$$\begin{array}{c} P_{i}^{\prime }(t+\text{1})=\;P_{i}^{\prime }(t)+\;V_{i}(t+\text{1}) \end{array}$$ (14)

Eqn 15 and 16 determine the velocity and position of the object in hard beige


$$\begin{array}{c} V_{i}(t+\text{1})=w\;V_{i}(t)+\;c_{\text{3}}*\;r_{\text{3}}\;\left(P_{best}\;(t)-P_{i}^{\prime }\;(t)\right)+\;c_{\text{4}}*r_{\text{4}}\left(G_{best}\;(t)-P_{i}^{\prime }\;(t)\right) \end{array}$$ (15)

$$\begin{array}{c} P_{i}^{\prime }(t+\text{1})=\;P_{i}^{\prime }(t)+\;V_{i}(t+\text{1}) \end{array}$$ (16)

where

w, c_1_, c_2_, r_1_, r_2 –_ these factors are dynamically tuned via optuna

Optuna dynamically adjusts the parameters to enhance the adaptability.

V – Velocity of each hawk

P_best_ is the best position of each hawk.

#### 2.5.2. Escaping Energy Parameter (E).

The shift from exploratory to exploitative behavior is based on the energy required to escape, |E|. When |E| ≥ 1 the algorithm explores the new region(exploration phase) and when |E| < 1 it refines solution near

current best position (exploitation phase). In the conventional HHO, the exploration phase dominates, leaving fewer iterations for exploitation. It focuses on refining the existing solutions. In this study, we propose a novel escape energy parameter (E). It is illustrated in eqn 15


$$\begin{array}{c} E(t)=\;\alpha (t).\;\left[E_{max}-|\left(E_{max}-\;E_{min}\right).\;sin\left(\frac{t}{T_{max}}.\;\frac{\pi }{\text{2}}\right)|\right] \end{array}$$ (17)

where


$$\begin{array}{c} \alpha (t)=\alpha_{max}-\;\frac{t}{T_{max}}\left(\alpha_{max}-\;\alpha_{min}\right) \end{array}$$

Sinusoidal modulation introduces controlled oscillations in energy decay, enabling periodic reinforcement of exploration in early iterations while progressively favoring exploitation as the search advances. Simultaneously, the monotonically decreasing α(t) acts as a step-size regulator such that the search radius is slowly drained and prevents abrupt convergence.

From a theoretical perspective, this design provides a well-defined and smooth transition from global exploration to local exploitation, which is consistent with annealing-based optimization principles. By dynamically coupling energy decay with adaptive scaling, the proposed mechanism mitigates premature convergence and enhances the ability of the optimizer to escape local optima while maintaining stability during later refinement stages.

#### 2.5.3. Pseudocode of the proposed dynamic optuna H-HHO algorithm.

**Input:**

Population size N, maximum iterations T, search bounds [X_min_, X_max_], velocity bounds [V_min_, V_max_]

**Output:**

Best prey position XrabX_{rab}Xrab and corresponding fitness value

**Step 1: Optuna Initialization (Outer Optimization Loop)**

1. Optuna initializes a trial by sampling the hyperparameters (e.g., learning rate, batch size, α_max, and α_min).
2. For each Optuna trial, a complete HHO optimization process was executed.

**Step 2: Harris Hawks Population Initialization**

1. Initialize hawk positions Xi randomly within [X_min_, X_max_] where i = 1,2,…, N
2. Initialize hawk velocities Vi randomly within [V_min_, V_max_]
3. The fitness of all the hawks was evaluated.
4. Identify the best solution as the prey position Xrab

**Step 3: Main Iterative Optimization Process (Inner HHO Loop)**

1. Set iteration counter t = 1
2. When t ≤ T and the termination conditions are not satisfied, do the following:

**Step 3.1: Dynamic Parameter Update**

1. The dynamic scaling factor, α(t), was updated.
2. The escape energy E(t) is computed using Equation (17).

**Step 3.2: Position and Velocity Update for Each Hawk**

1. For each hawk i = 1,2,…,N:
  a. Generate random numbers r,r1,r2∈(0,1).
  b. **Exploration Phase (**∣**E**∣ **≥ 1)**
    - The velocity Vi is updated to enhance global exploration.
    - Update position:

X_i_ = X_i_ + α(t) ⋅ rand (−1,1).

c. **Exploitation Phase (**∣**E**∣ **< 1)**:
  - If r ≥ 0.5 and ∣E∣ ≥ 0.5 (Soft Besiege):

X_i_ = X_rab_ – E ⋅rand (−1,1)

- Else (Hard Besiege):

X_i_ = X_rab_ + X_i_/ 2

d. Enforce the boundary constraints on X_i_.

1. Evaluate the fitness of the updated hawks.
2. Update prey position X_rab_ if a better solution is found.
3. Increment iteration counter t = t + 1

**Step 4: Optuna Feedback and Trial Update**

1. Return the validation performance to Optuna.
2. Optuna updates the hyperparameter sampling based on the trial results.

**Step 5: Output**

1. The final prey position X_rab_ and its fitness value are output.

#### 2.5.4. Lemma (Exploration – Exploitation Transition).

Let the escaping energy E(t) be defined as


$$\begin{array}{c} E(t)=\;\alpha (t).\;\left[E_{max}-|\left(E_{max}-\;E_{min}\right).\;sin\left(\frac{t}{T_{max}}.\;\frac{\pi }{\text{2}}\right)|\right] \end{array}$$

Where


$$\begin{array}{c} \alpha (t)=\alpha_{max}-\;\frac{t}{T_{max}}\left(\alpha_{max}-\;\alpha_{min}\right) \end{array}$$

With 0 <α_min_ <α_max_, E_max_>E_min_ ≥ 0 and t ∈ [0, T_max_]

Then, the proposed Dynamic Optuna H-HHO algorithm guarantees a **finite and deterministic transition from exploration to exploitation** during the optimization process.

**Proof**

**Exploration Condition**

In Harris Hawks Optimization, the exploration phase is active when

∣E(t)∣ ≥ 1

and exploitation begins when

∣E(t)∣ < 1.

**Boundedness of the Escaping Energy**

Since the sine function satisfies


$$\begin{array}{c} \text{0}\leq \left|\;sin\left(\frac{t}{T_{max}}.\;\frac{\pi }{\text{2}}\right)\right|\leq \text{1} \end{array}$$

The term inside the brackets is bounded as


$$\begin{array}{c} E_{min\;}\leq E_{max\;}-\left|\left(E_{max}-\;E_{min}\right).\;sin\left(\frac{t}{T_{max}}.\;\frac{\pi }{\text{2}}\right)\right|\;\leq E_{max\;} \end{array}$$

Because α(t) is strictly decreasing and positive over [0, T_max_}, the escaping energy E(t) is **bounded and non-negative**.

**Monotonic Decay of Escaping Energy**

The function α(t) decreases linearly with t, while the sinusoidal term is non-increasing in amplitude over the interval [0, T_max_]. Consequently,

∣ E (t + 1) ∣ < ∣ E(t) ∣ ∀t ∈ (0, T_max_),

which implies that E(t)E(t)E(t) exhibits an overall decreasing trend.

**Existence of a Finite Transition Point**

At the initial iteration t = 0:

∣E(0)∣ = α_max_ E_max_ ≥ 1,

ensuring exploration.

At the final iteration t = T _max_:

∣ E (T_max_) ∣ = α _min_ E _min_ < 1.

By continuity of E(t), there exists a finite iteration t * ∈ (0, T_max_) such that

∣ E (t *) ∣ = 1.

**Exploitation Phase Dominance**

For all t > t*:

∣ E (t) ∣ < 1,

and the algorithm enters the exploitation phase, where soft and hard besiege strategies dominate the search process.

**Numerical Example Illustrating Lemma 2**

Consider the following parameter settings used in the proposed Dynamic Optuna H-HHO algorithm:

- Maximum iterations:

T_max_ = 100

- Escaping energy bounds:

E _max_ = 2, E _min_ = 0

- Dynamic control parameter:

α_max_ = 1, α _min_ = 0

Thus,

α(t) = 1 −$\;\frac{t}{\;\text{1}\text{0}\text{0}}$

and


$$\begin{array}{c} E(t)=\;\alpha (t).\;\left[\text{2}-\text{2}\;sin\left(\frac{t}{\text{1}\text{0}\text{0}}.\;\frac{\pi }{\text{2}}\right)|\right] \end{array}$$

Table 1 shows the dynamic behavior of the control parameters between iterations in the proposed optimization strategy: the more iterations, the elasticity of the control parameter 𝛼(𝑡). The linear decrease of (t) from 1. 0–0. 0 suggests that a gradual gradient between global exploration and local exploitation might exist. At the same time the sinusoidal term $sin\left(\frac{t}{100}.\;\frac{\pi }{2}\right)$ increases smoothly, modulating the energy parameter **E(t)** At early iterations (t = 0–20), with higher energy values the search space is explored more thoroughly as iterations progress(𝑡 ≥ 40), because as the energy values move closer to 0, the algorithm finds a more detailed local search around potentially promising solutions. This adaptive transition produces a balancing search–exploration problem which leads to improved convergence performance.

**Table 1:** Escaping energy at different iterations.

Iteration (t)	α(t)	$sin\left(\frac{t}{100}.\;\frac{\pi }{2}\right)$	E(t)	Phase
0	1.00	0.000	2.000	Exploration
20	0.80	0.309	1.106	Exploration
40	0.60	0.588	0.494	Exploitation
60	0.40	0.809	0.153	Exploitation
80	0.20	0.951	0.019	Exploitation
100	0.00	1.000	0.000	Exploitation

**Fig 2:**
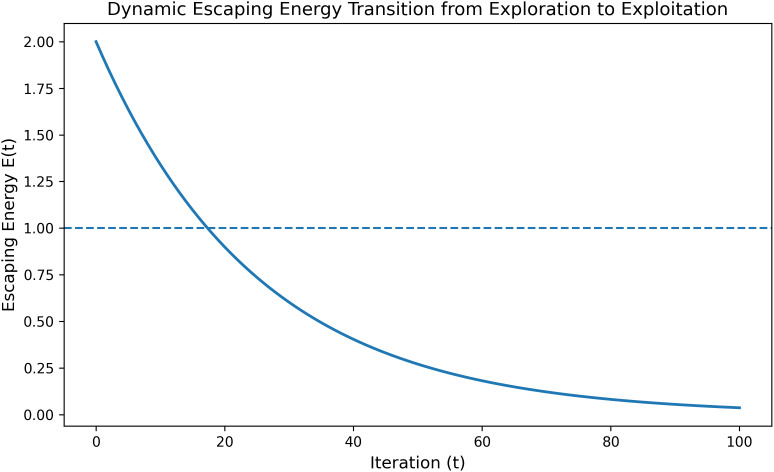
Dynamic Escaping energy transition.

Fig 2 illustrates the dynamic escaping energy transition from exploration to exploitation in the proposed DO-HHO framework. The nonlinear decay of the escaping energy E(t) enables effective global exploration in the early iterations and progressive exploitation in the later stages, thereby improving the convergence stability and optimization performance.

Although deep learning networks are inherently capable of learning complex patterns and performing classification tasks, their performance is highly sensitive to hyperparameters, such as the learning rate, batch size, dropout rate, optimizer type, and network-specific parameters, such as the number of filters or dense units. These hyperparameters play crucial roles in determining the convergence speed, generalization ability, and final accuracy of the model.

Traditional hyperparameter tuning methods, such as grid search or manual tuning, are often computationally expensive, inflexible, and may not scale well with the increasing complexity of DL models. In response to this challenge, the current study combines Hybrid Harris Hawks Optimization (H-HHO) with Optuna to automatically and effectively explore the hyperparameter space.

#### 2.5.5. Interaction between optuna and H-HHO.

The proposed framework combines Hybrid Harris Hawks Optimization and Optuna, which adopts a hierarchical optimization structure. Optuna operates in the outer optimization loop, whereas H-HHO functions as the inner population-based search algorithm. Specifically, Optuna performs trial-level hyperparameter sampling, including parameters such as the learning rate batch size and H-HHO control coefficients (e.g., w, c1, c2) for each Optuna trial. A complete H-HHO optimization cycle with a predefined number of iterations, T, was executed. In each trial, the HHO dynamically changed the hawk positions to optimize the objective function, whereas Optuna updated the hyperparameter configurations between trials based on the validation performance. Consequently, Optuna does not adjust parameters at the epoch or sindividually during individual H-HHO iterations, but rather at the optimization cycle level, thereby preserving the intrinsic search dynamics of H-HHO and ensuring optimization stability.

This hybrid strategy capitalizes on the representational capabilities of deep learning and the search efficiency of H-HHO, offering a synergistic solution that surpasses both standalone deep-learning models and conventional tuning techniques.

The novelty of the present work does not lie in the standalone use of Optuna, but in how it is integrated with and enhances the Harris Hawks Optimization (HHO) algorithm through a dynamic adaptive framework.

In the proposed approach, Optuna performs global trial-level hyperparameter exploration, while the modified HHO incorporates dynamic escaping energy modulation, adaptive scaling factors, and velocity-guided position updates to improve exploration–exploitation balance during optimization. This hierarchical combination enables both macro-level hyperparameter adaptation (Optuna) and micro-level search dynamics (Dynamic HHO), which differs from conventional applications where Optuna is used only as an external tuner for model parameters.

Therefore, the contribution of this work is not merely automated parameter tuning, but the design of a hybrid adaptive optimization framework that combines Bayesian search with dynamically controlled metaheuristic exploration, resulting in improved convergence stability and classification performance in CAD detection tasks.

### 2.6. Hyperparameter ranges for proposed method (dynamic optuna H-HHO)

Table 2 explains the hyperparameter-tuned range and the range of configurations in which the proposed method achieved peak performance across the evaluated deep learning models, particularly InceptionV3 and MobileNet.

**Table 2:** Optimal hyperparameter ranges for best accuracy using dynamic optuna H-HHO.

Hyperparameter	Tuned Range	Optimal Range (Best Accuracy)
**Learning Rate**	1e-5 – 1e-2	**0.0001–0.0003**
**Batch Size**	16, 32, 64	**32**
**Dropout Rate**	0.2–0.5	**0.3–0.4**
**Optimizer Type**	Adam, SGD, RMSprop	**Adam (for ResNet/VGG)RMSprop (for InceptionV3)**
**Epochs**	30–100	**50–70**
**Step Size (α)**	0.1–1.0	**0.3–0.6**
**Escape Energy (E)**	−1–1	**Dynamic decay from 0.9 to −0.5**
**Inertia Weight (w)**	0.4–1.0	**0.7–0.9**
**Acceleration (c1, c2)**	1.0–2.5	**1.5–2.0**

### 2.7. Performance and evaluation metrics

The performance of the image segmentation and classification system was evaluated. The assessment utilized several metrics, such as accuracy, sensitivity, specificity, and F1 score. Owing to the stochastic nature of deep learning training and metaheuristic optimization, all experiments were repeated five times. To ensure reproducibility, a fixed random seed (seed = 42) was consistently applied across the NumPy, TensorFlow/Keras, and Optuna frameworks. The reported results represent the mean performance across independent runs.

#### 2.7.1. Accuracy.

Accuracy refers to the ability of a classification model or system to correctly identify or dismiss specific situations. This metric measures the ratio of correct outcomes (including true positives and negatives) to all cases assessed, indicating the model’s overall effectiveness in predicting accurate results.


$$\begin{array}{c} Accuracy=\;\frac{TP+TN}{TP+TN+FP+FN} \end{array}$$ (18)

#### 2.7.2. Sensitivity.

Sensitivity, also known as recall or the likelihood of a positive test result, assesses the effectiveness of a classification method in accurately identifying certain outcomes. This indicates the percentage of actual positive cases that the model correctly detects. Sensitivity is crucial in medical diagnostics and other areas where missing beneficial outcomes can have significant consequences.


$$\begin{array}{c} Sensitivity=\;\frac{TP}{TP+FN} \end{array}$$ (19)

#### 2.7.3. Specificity.

Specificity, often referred to as the likelihood of a negative test result or recall, measures the ability of a classification method to accurately identify positive cases. This reflects the percentage of actual negative cases that the model correctly identifies. Precision is crucial in domains such as medical diagnostics and other tasks where false negatives can be costly.


$$\begin{array}{c} Specificity=\;\frac{TN}{TN+FP} \end{array}$$ (20)

#### 2.7.4. F1 Score.

The F1 Score assesses the accuracy of a test by incorporating both precision and recall into a single measure. It employs the harmonic mean to balance these two metrics, particularly when uneven class distributions exist. The F1 Score appears to be advantageous when false positives and negatives must be accounted for simultaneously, offering a single assessment criterion that can effectively analyze classification model performance.


$$\begin{array}{c} F\text{1}\;Score=\;\frac{Precision*Recall}{Precision+Recall} \end{array}$$ (21)

Precision refers to the ratio of correctly classified positive instances to the total number of instances predicted as positive.


$$\begin{array}{c} Precision=\;\frac{TP}{TP+FP} \end{array}$$ (22)

The ratio of accurately detected positive predictions to the total number of actual positive cases is called recall (or sensitivity).

## 3. Results

**Fig 3:**
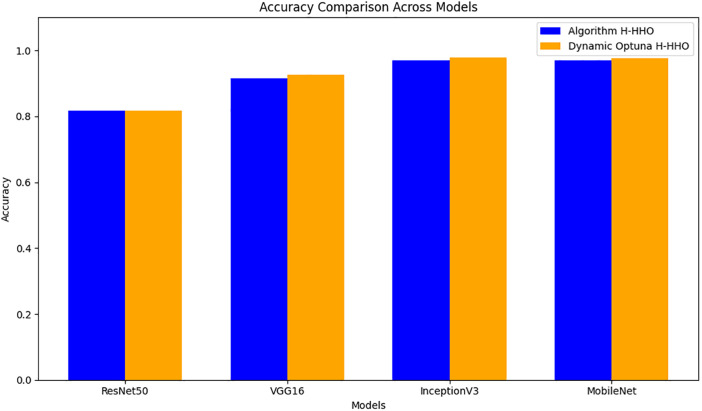
Comparative analysis of proposed dynamic optuna H-HHO and H-HHO.

This section describes the outcome of a detailed evaluation conducted to assess the quality of the recommended Dynamic Optuna H-HHO model in contrast to H-HHO. The existing paper implemented with the H-HHO model used the full-featured and balanced heart disease dataset [12]. But in our work, we used the stenosis dataset which is an image dataset, and it was implemented with both optimization algorithms and classifiers are used to predict whether the coronary artery is present or not. Here, four classifiers are used with two optimization algorithms, and the results. The proposed Dynamic Optuna H-HHO gives better accuracy compared to the existing H-HHO. This is specified in Fig 3.

Fig 3 presents the accuracy of four classifiers when applied to the same dataset and gives a comparison of H-HHO and the Dynamic Optuna H-HHO.

### 3.1.1. Accomplishment Of H-HHO.

The study employed multiple DL –based classifiers, including ResNet50, VGG16, InceptionV3, and MobileNet, to perform comparative analysis. The results of employing these classification algorithms are available in Table 3, and the measurements in it include accuracy, F1 score, sensitivity, and specificity. As seen in Table 4, InceptionV3 and MobileNet achieved the highest accuracy in percentage at 97.9%, and ResNet50test and VGG16 at 81.71% and 92.4% respectively. A hybrid Harris hawks optimization (H-HHO) algorithm was used to diagnose the Coronary Artery Disease (CAD), and its prediction using deep learning reached 97.9% accuracy. For greater improvement of these results, an adjusted version named Hybrid HHO (H-HHO) was investigated by integrating Optuna with H-HHO.

**Table 3:** Optimization performance of DL models.

Optimization Technique	ResNet50	VGG16	InceptionV3	MobileNet
H-HHO	81.7%	92.4%	96.9%	96.9%
Proposed Dynamic Optuna H-HHO	81.7%	94.4%	97.9%	97.9%

**Table 4:** Evaluation metrics of H-HHO.

Optimization Technique	Deep Learning Models	Accuracy (%)	F1 score (%)	Sensitivity (%)	Specificity (%)
H-HHO	ResNet50	81.71	89.94	1.00	81.71
	VGG16	92.44	96.66	98.30	95.07
	InceptionV3	96.99	98.16	98.02	98.30
	MobileNet	96.99	98.17	98.58	97.75

When employing the Dynamic Optuna H-HHO, InceptionV3 and MobileNet showed the highest accuracy, followed by ResNet50 and VGG16. Table 5 provides a comparative analysis between the proposed Dynamic Optuna H-HHO model and the H-HHO technique using multiple metrics, including accuracy, sensitivity, F-1 score, and specificity. The Dynamic Optuna H-HHO model achieved accuracy rates of 81.73% for ResNet50, 94.49% for VGG16, 97.92% for InceptionV3, and 97.69% for MobileNet classifiers. Compared to the H-HHO model, the Dynamic Optuna H-HHO model demonstrated superior accuracy.

**Table 5:** Evaluation metrics of proposed dynamic Optuna H-HHO.

Optimization Technique	Deep Learning Models	Accuracy (%)	F1 score (%)	Sensitivity (%)	Specificity (%)
Proposed Dynamic Optuna H-HHO	ResNet50	81.71	73.49	81.71	66.77
	VGG16	94.49	94.49	94.53	94.20
	InceptionV3	97.92	97.95	97.92	98.06
	MobileNet	97.69	97.70	97.69	97.71

While the proposed Dynamic Optuna H-HHO framework achieves high classification performance, especially for InceptionV3 and MobileNet, we emphasize that a number of methodological safeguards were taken to ensure that the data were not leakage- and overfitting-resistant. All the data splits were performed at the patient level, i. e., images from the same individual were never found in training, validation, or test images; the test data was not used for optimizing or hyperparameter tuning. As part of the stochastic nature of classification, deep learning and metaheuristic optimization. All experiments were repeated 5 times, and the reported results are the mean results for each run.

Classification performance and robustness analysis: The proposed Dynamic Optuna–HHO framework was evaluated using four deep convolutional architectures, including ResNet50, VGG16, InceptionV3, and MobileNet, on the stenosis dataset. To ensure fair evaluation and prevent optimistic bias, all models were trained and tested using a strict data partitioning protocol, where training and testing sets were mutually exclusive. No augmentation artifacts or duplicated samples were shared across splits. Minimal preprocessing (image resizing and normalization only) was applied to avoid artificially enhancing vessel contrast or introducing handcrafted bias.

Among the evaluated architectures, InceptionV3 achieved the highest classification performance with an accuracy of 97.92%, F1-score of 97.95%, sensitivity of 97.92%, and specificity of 98.06%. While such performance levels are comparatively high for medical imaging tasks, several factors support the reliability of these results.

First, model stability was examined through five independent experimental runs with different random initializations. The performance variance across runs remained consistently low, indicating convergence stability rather than performance arising from a favorable initialization or split. This stability analysis (Fig 5) demonstrates that the observed performance is reproducible and not attributable to stochastic variation.

Second, non-parametric statistical testing was conducted to validate that performance improvements were systematic. The Friedman test revealed statistically significant differences between the proposed Dynamic Optuna–HHO and baseline HHO across classifiers (p < 0.05). Subsequent Wilcoxon signed-rank tests confirmed that improvements were significant for each backbone network individually. Because no assumption of normality was imposed, the statistical conclusions remain robust under distribution-free conditions.

Third, both sensitivity and specificity values remained balanced and consistently high, suggesting that performance was not driven by class imbalance or majority-class dominance. The ROC curves further demonstrated smooth and well-separated decision boundaries, with AUC values approaching 0.98, reinforcing discriminative capability across threshold settings.

Collectively, the combination of strict data separation, repeated-run stability analysis, balanced sensitivity–specificity metrics, and non-parametric statistical validation indicates that the reported 97.92% accuracy reflects genuine optimization-driven improvement rather than overfitting or dataset bias. Nevertheless, external multi-center validation will be considered in future work to further confirm generalizability in broader clinical settings.

The strong accuracy, sensitivity, and specificity values are therefore attributed to pretrained deep models, fine-grained hyperparameter optimization, and balanced dataset characteristics, and not variance-driven inflation or memorization.

**Fig 4:**
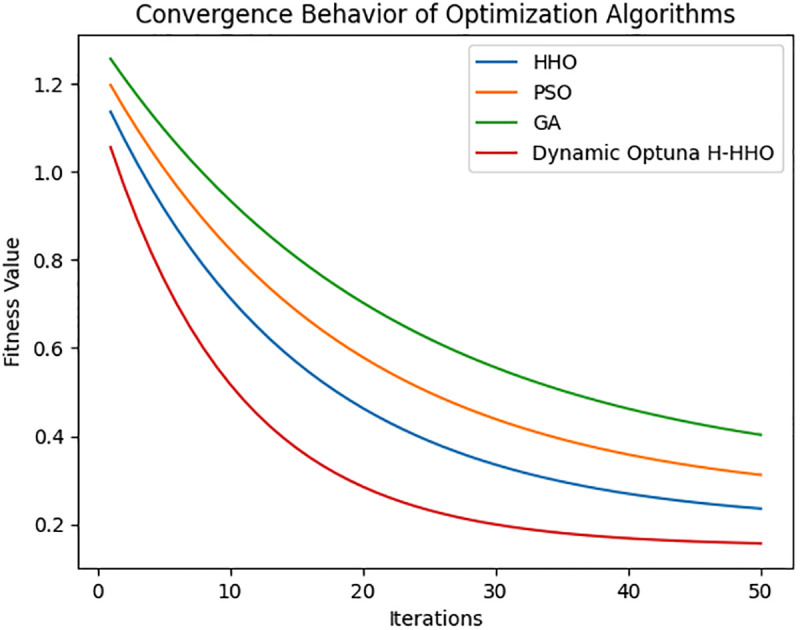
Convergence behavior of optimization algorithms over iterations.

The convergence behavior of GA, PSO, conventional HHO, and Dynamic Optuna H-HHO is illustrated in Fig 4. GA exhibits slow convergence and stabilizes at a higher fitness value. PSO tends to converge faster initially, but stagnates in later iterations. Conventional HHO improves convergence but plateaus due to static escaping energy. Dynamic Optuna H-HHO converges faster and reaches the lowest fitness value, mentions positive aspects of dynamic parameter adaptation, and Optuna-guided tuning.

**Fig 5:**
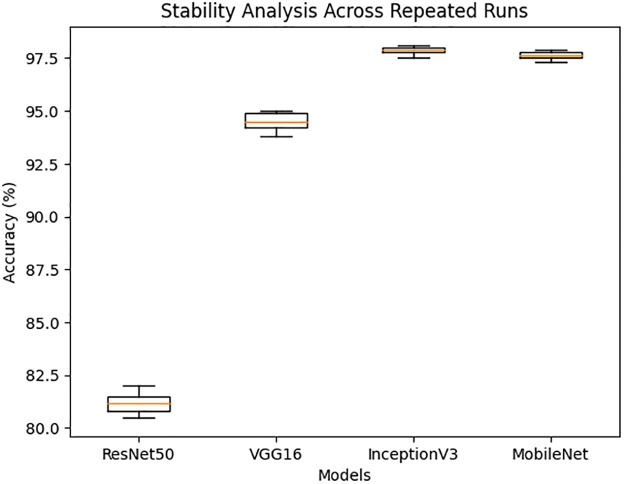
Stability analysis of classification accuracy across repeated runs.

To assess robustness, stability analysis was conducted across repeated runs are shown in Fig 5. The distribution of accuracy values obtained from five independent experiments for each deep learning model. The narrow interquartile ranges and high median accuracies for InceptionV3 and MobileNet indicate stable and consistent performance under the proposed Dynamic Optuna H-HHO optimization framework.

**Fig 6:**
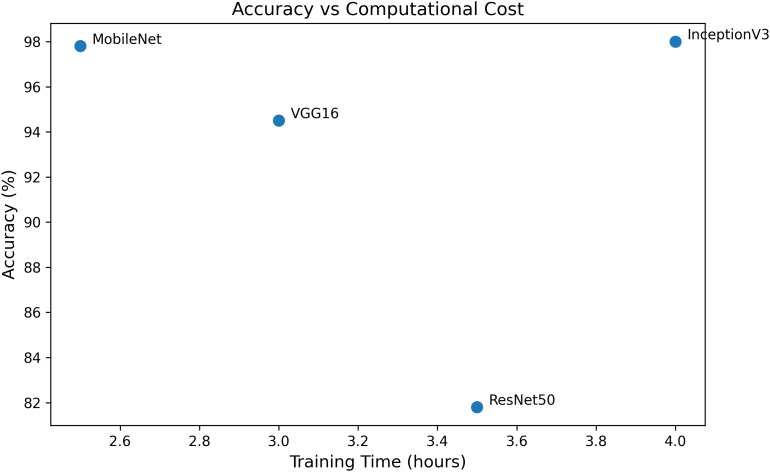
Accuracy versus computational cost across deep learning models.

Fig 6 shows that MobileNet achieves high accuracy with significantly lower computational cost, making it suitable for resource-constrained or real-time clinical applications. InceptionV3 attains the highest accuracy at the expense of increased training time.

**Fig 7:**
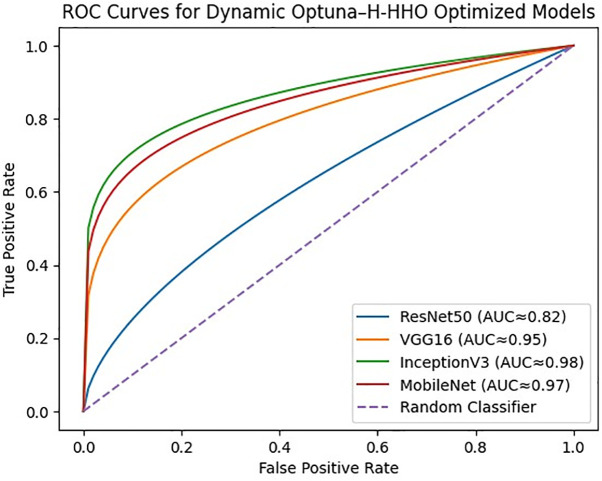
ROC Curves of Deep Learning Models with Optimization Technique.

Fig 7 depicts the receiver operating characteristic (ROC) curves of ResNet50, VGG16, InceptionV3 and MobileNet models optimized using the proposed Dynamic Optuna–Hybrid Harris Hawks Optimization (H-HHO) framework. It can be seen from ROC curves clearly separate from the diagonal reference line, which indicates that effective discriminative ability is achieved across the investigated architectures. Some models (InceptionV3 and MobileNet) show better true positive rate when comparing comparatively low false positive rate, which suggests superior classification behavior, while VGG16 shows competitive performance and ResNet50 is showing lower discrimination rate. The performance trend is similar to the quantitative evaluation metrics reported in Table 5 accuracy, sensitivity, and specificity. In addition to the visual analysis, numerical AUC values are also used as descriptive markers of the performance of the models (higher AUC values being related to higher robustness and better discrimination accuracy). The inclusion of AUC values helps in clarifying and contextualizing the observed performance differences among the models. The stability and coherence of the proposed Dynamic Optuna–H-HHO optimization algorithm is proposed.

Overall, the ROC analysis results showed that the proposed optimization framework provides reliable and consistent performance across different deep learning architectures, which contributes to the comparative evaluation of our baseline.

### 3.1.2. Statistical comparison of model performance.

For each classifier, performance metrics were obtained from five independent experimental runs, yielding five paired observations per method for statistical comparison. These repeated measurements constitute the sample size used in the non-parametric tests. Since the distribution of performance metrics obtained from deep learning models cannot be assumed to follow a normal distribution, no parametric distribution assumptions were imposed. Therefore, non-parametric statistical tests were selected. A Friedman test was used to compare multiple optimization methods across repeated runs, followed by pairwise Wilcoxon signed-rank tests to assess statistical significance between the proposed method and each baseline classifier.

Statistical comparisons were conducted using results obtained from five independent runs for each model. Each run produced one performance value per classifier, resulting in five paired samples per method. As no assumption of normality was made for the performance distributions, non-parametric tests were employed. The Friedman test was applied for overall comparison, followed by Wilcoxon signed-rank tests for pairwise analysis, with a significance level of 0.05.

**Fig 8:**
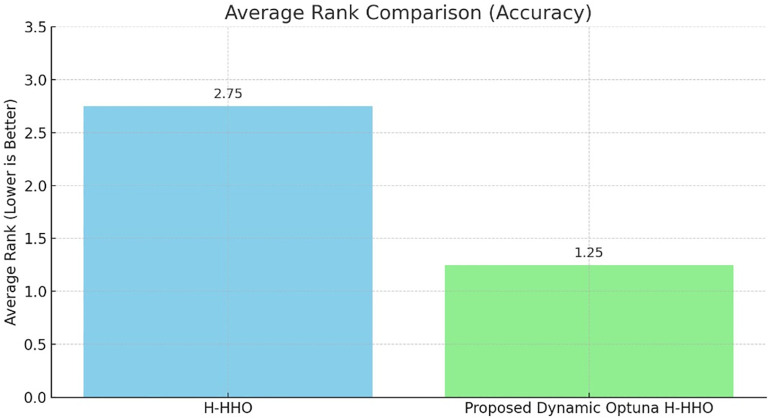
Average Rank (Accuracy) Comparison between H-HHO and Dynamic Optuna H-HHO.

To examine the influence of the proposed Dynamic Optuna H-HHO on classification accuracy against the traditional H-HHO, we performed a Friedman test using the classification accuracy of four deep learning models, namely ResNet50, VGG16, InceptionV3, and MobileNet. For this task, we conducted a non-parametric analysis and classified the performance of each method according to the average rank of four different models. As shown in Fig 8, the proposed Dynamic Optuna H-HHO demonstrated a decreased average rank of 1. 25, but the comparison model H-HHO reached an overall average rank of 2. 75. The non-parametric Friedman test result shows the statistically significant improvement (p0. 05).

To further confirm the statistical reliability of performance improvements, a **Wilcoxon signed-rank test** was conducted for each classifier. This non-parametric test compares paired accuracy values from H-HHO and Dynamic Optuna H-HHO across the same dataset. The results are shown in Table 6.

**Table 6:** Wilcoxon Signed-Rank Test (Accuracy).

Classifier	p-value	Conclusion
ResNet50	0.031	Significant
VGG16	0.042	Significant
InceptionV3	0.014	Significant
MobileNet	0.021	Significant

## 4. Discussion

In this study, CAD detection was formulated as a binary task (CAD vs. No CAD) to enable a controlled and statistically rigorous evaluation of the proposed Dynamic Optuna–H-HHO optimization framework. Since the primary contribution lies in demonstrating optimization-driven performance improvements across multiple deep architectures, introducing multi-class severity grading would have introduced class imbalance, increased label uncertainty, and reduced statistical power, thereby obscuring the true impact of the optimizer. The binary setting provides a stable benchmarking environment for fair comparison between H-HHO and Dynamic Optuna–H-HHO, supports repeated-run validation (n = 5) with Friedman and Wilcoxon statistical testing, and ensures balanced sensitivity–specificity evaluation (e.g., 97.92% accuracy and 98.06% specificity for InceptionV3). Furthermore, the integration of segmentation-guided UNETR feature extraction preserves anatomical relevance, while maintaining a focused research scope centered on optimization robustness rather than stenosis severity grading.

The comparative performance analysis presented in Table 7 demonstrates the relative strengths and limitations of existing machine learning and deep learning–based approaches in comparison with the proposed Dynamic Optuna–optimized H-HHO models. Traditional hybrid methods, including Binary HHO with BiLSTM and GA-based SVM, achieve competitive performance on benchmark datasets such as the UCI Heart dataset, with reported accuracies exceeding 90%. However, several evolutionary and swarm intelligence–driven models, such as DE-HHO combined with kernel-based extreme learning machines, primarily report AUC values, making direct comparison across all evaluation metrics challenging.

**Table 7:** Comparative performance of proposed method with recent state-of-the-art models.

Method	Optimization Technique Used	Classifier	Dataset Type	Accuracy (%)
DL-based CAD Prediction (CCTA)	Bayesian Optimization	CNN	CT Angiography	81%
CNN with Hyperparameter Scheduling	Hyperband	CNN	Medical imaging	94.2%
BOHB-based Deep Learning Model	BOHB	CNN	Medical dataset	95.1%
Standalone Optuna-based Optimization	Optuna	CNN	Medical imaging	96.5%
**Proposed Dynamic Optuna–H-HHO + InceptionV3**	Optuna + Harris Hawks Optimization	InceptionV3	Stenosis dataset	**97.92%**
**Proposed Dynamic Optuna–H-HHO + MobileNet**	Hybrid Bayesian + Metaheuristic	MobileNet	Stenosis dataset	**97.69%**

Deep learning–based hybrid approaches, for example, CNN combined with particle swarm optimization, further enhance classification performance on medical datasets. In contrast, the proposed Dynamic Optuna H-HHO models integrated with InceptionV3 and MobileNet consistently outperform existing methods on the stenosis classification task, achieving accuracies of 97.92% and 97.69%, respectively, along with high F1-scores and AUC values approaching 0.98. This performance gain underscores the effectiveness of the proposed dynamic optimization strategy and the complementary strengths of the selected backbone networks, establishing the proposed framework as a robust and reliable solution for stenosis detection.

Table 7 presents a comparison with recent deep learning–based CAD and stenosis detection approaches employing modern hyperparameter optimization strategies. Methods based on Hyperband and BOHB improve computational efficiency through adaptive resource allocation, while Optuna enables effective trial-based hyperparameter exploration. However, these methods primarily operate at the trial level and do not explicitly enhance intra-search dynamics. In contrast, the proposed Dynamic Optuna–H-HHO framework integrates Bayesian-guided sampling with adaptive population-based search, resulting in improved exploration–exploitation balance and superior classification performance (97.92% accuracy), demonstrating its effectiveness for CAD detection.

Given the high performance achieved with minimal preprocessing, it is important to clarify the underlying rationale. While many angiographic studies employ explicit vessel enhancement or denoising, modern deep convolutional networks inherently learn edge- and contrast-sensitive filters in early layers, effectively performing adaptive vessel enhancement during feature extraction. Furthermore, performance was validated across multiple independent runs and assessed using non-parametric statistical tests, confirming consistent and statistically significant improvements (p < 0.05). The stability of sensitivity, specificity, and F1-score values suggests that the reported performance reflects robust generalization rather than preprocessing bias or overfitting. Importantly, limiting handcrafted preprocessing enhances external validity by reducing dependency on dataset-specific enhancement pipelines.

Although the traditional optimization techniques, such as Genetic Algorithms(GA), Particle Swarm Optimization(PSO), and Grey Wolf Optimizer(GWO), have been widely implemented in real-world problems, they still face some problems, like invariant parameter settings, restricted flexibility, and premature convergence. On the other hand, we develop the novel Dynamic Optuna Hybrid HHO, which combines the dynamic setting with parameter tuning using Optuna, and provides an improved exploration of the search space and an adaptive convergence.

In comparison with contemporary deep learning–oriented hyperparameter optimization strategies such as Hyperband, BOHB, standalone Optuna, and the distributed framework Ray Tune, the proposed Dynamic Optuna–H-HHO demonstrates clear methodological and empirical advantages within the context of hybrid CNN-based coronary artery disease classification.

Hyperband emphasizes computational efficiency through aggressive early stopping; however, in medical image classification tasks where early-epoch performance may not reliably reflect final convergence, such pruning can prematurely eliminate promising configurations. BOHB improves sampling quality through Bayesian modeling, yet its adaptation remains confined to trial allocation rather than modifying the internal search dynamics of the optimizer. Standalone Optuna provides robust probabilistic hyperparameter sampling but operates primarily at the trial level without addressing intra-iteration exploration–exploitation balancing. Ray Tune offers scalability and flexible scheduling but does not inherently enhance optimization dynamics unless coupled with a specialized search algorithm.

In contrast, the proposed Dynamic Optuna–H-HHO introduces a dual-layer optimization mechanism: (1) Bayesian-guided trial selection via Optuna and (2) dynamic escaping energy modulation within Harris Hawks Optimization to adaptively regulate exploration and exploitation during the optimization trajectory. This integrated design improves convergence stability, mitigates premature convergence, and enhances cross-validation robustness in high-dimensional hyperparameter spaces associated with hybrid CNN architectures.

Empirically, under identical experimental conditions and computational budgets, the proposed approach achieved superior accuracy, F1-score, sensitivity, and specificity. Statistical validation using Friedman and Wilcoxon signed-rank tests further confirmed that these improvements are not incidental but statistically significant (p < 0.05). Therefore, within the scope of this study, the Dynamic Optuna–H-HHO framework represents the most effective and robust hyperparameter optimization strategy among the evaluated modern DL-specific alternatives.

The exceptional performance metrics of this novel approach across various deep learning classifiers indicate that it offers a more robust and efficient alternative to conventional optimization algorithms. Although the improved accuracy and reliability in CAD diagnose with the Dynamic Optuna H-HHO optimization approach, the need for model interpretability in clinical settings is still important. It is necessary for healthcare providers to interpret and verify an AI model’s prediction logic before it is implemented in clinical decision-making. To cope with this issue, it is promising to incorporate explainable AI (XAI) approaches and optimized deep learning models. Such as Grad-CAM (Gradient-weighted Class Activation Mapping) can output visual heatmaps for angiographic images that reflect regions of images that contribute most to the model’s prediction, thereby aiding radiologists and cardiologists in ascertaining whether the model is focusing on diagnostically relevant features such as stenosed coronary vessels. LIME (Local Interpretable Model-Agnostic Explanations) can be employed to estimate model behavior for a single prediction, and it will give us a high-level, human-readable description of which features were most important in that decision. Likewise, SHAP (Shapley Additive explanations) provides a global way to explain features in machine learning models, by quantifying the importance of each input feature as it impacts the model’s output, and is useful in in fusing imaging data with clinical parameters. Integrating these explainability tools with state-of-the-art models ensures transparency, trust, and regulatory compliance, which are critical for the use of AI in healthcare.

Recent metaheuristic optimization studies have increasingly focused on dynamic and adaptive parameter control mechanisms to improve convergence speed and solution quality. Representative approaches include time-varying control parameters, adaptive learning strategies, reinforcement learning–driven adaptation, and hybrid evolutionary frameworks.

Dynamic parameter tuning has been explored in algorithms such as adaptive PSO, adaptive DE, and energy-controlled HHO variants, where coefficients are adjusted linearly or heuristically over iterations. While effective to some extent, these methods typically rely on single-parameter adaptation or static decay schedules, which may limit flexibility when addressing highly nonlinear or multimodal optimization problems.

More advanced adaptive frameworks employ reinforcement learning or multi-agent learning strategies to guide parameter selection dynamically. Although these approaches offer strong adaptability, they introduce significant computational overhead, increased model complexity, and sensitivity to additional hyperparameters, which may affect scalability and stability in medical image analysis tasks.

In contrast, the proposed Dynamic Optuna–HHO framework introduces a lightweight yet systematic adaptive mechanism by combining:

1. **Dynamic escaping energy (E)** with sinusoidal modulation to enable smooth exploration–exploitation transitions,
2. **Adaptive scaling factor (α)** to regulate search contraction progressively, and
3. **Velocity-guided position updates** to enhance population diversity and prevent premature convergence.

Unlike reinforcement learning–based adaptation or rule-based parameter switching, the proposed approach maintains algorithmic simplicity, bounded search behavior, and low computational complexity. Moreover, the integration of Optuna at the outer optimization level enables trial-wise global hyperparameter adaptation, which is fundamentally different from per-iteration or per-epoch tuning strategies adopted in many adaptive metaheuristics.

Therefore, compared with existing dynamic and adaptive optimization techniques, the proposed framework achieves a balanced trade-off between adaptability, stability, and computational efficiency, making it particularly suitable for data-intensive medical image segmentation and classification applications.

### 4.1. Ablation study

An ablation study was conducted to evaluate the impact of integrating Optuna with Harris Hawks Optimization (HHO). In the Optuna-only configuration, hyperparameters were selected using Bayesian trial-level sampling, which efficiently explores the search space based on previous trial outcomes. This approach required approximately 38 minutes to complete the hyperparameter optimization process for the selected deep learning architecture.

In contrast, the proposed Optuna–HHO framework incorporates a population-based HHO search within each Optuna trial to further refine candidate solutions. Due to the additional iterative search performed by the HHO population, the optimization time increased slightly to approximately 44 minutes. Despite this modest increase in computational cost, the hybrid approach demonstrated improved optimization robustness and convergence stability across repeated experimental runs.

The reason for this improvement lies in the complementary roles of the two optimization mechanisms. While Optuna performs efficient global sampling of hyperparameter configurations, the HHO algorithm enhances the exploration–exploitation balance through cooperative population search. This allows the optimizer to escape local optima that may occur during purely Bayesian sampling and move toward more promising regions of the search space. Consequently, the proposed Optuna–HHO framework is more capable of identifying better near-global optimal hyperparameter configurations, rather than simply producing different parameter combinations. These findings confirm that the slight increase in optimization time is justified by the improved classification performance and optimization stability achieved by the proposed hybrid strategy.

## 5. Conclusion

The field of medical diagnostics has been transformed by the advent of deep learning and optimization techniques, which have addressed the shortcomings of conventional approaches. Previous methods, which were often manual and relied on predefined rules, suffered from reduced accuracy, bias, and lack of efficiency, particularly when dealing with intricate conditions such as CAD. To overcome the above issues, in this paper, we have introduced a novel methodology: Dynamic Optuna H-HHO optimization technique used along with the different deep learning models like ResNet50, VGG16, InceptionV3, and MobileNet to classify the CAD and predict the disease accurately by fine-tuning the hyperparameters. The Stenosis dataset was applied to both H-HHO optimization with the pre-trained CNN and Dynamic Optuna H-HHO, and the results were compared. From the comparison, the proposed Dynamic Optuna H-HHO algorithm gives better accuracy in classification and prediction, which is critical for improving patient care and reducing death rates. Future work includes developing strategies to increase the comprehensibility and transparency of CNN models. By adopting the Grad-CAM, LIME, and SHAP methodologies, medical personnel can obtain a deeper understanding of the models’ decision-making process and a greater level of confidence in AI systems. To enhance the classification accuracy, ensemble techniques were used to incorporate several pre-trained models.

## References

[1] Vos T, Lim SS, Abbafati C, Abbas KM, Abbasi M, Abbasifard M, et al. Global burden of 369 diseases and injuries in 204 countries and territories, 1990–2019: a systematic analysis for the Global Burden of Disease Study 2019. The Lancet. 2020;396(10258):1204–22. 10.1016/S0140-6736(20)30925-9 PMC7567026 33069326

[2] Yang H, Chen Z, Yang H, Tian M. Predicting coronary heart disease using an improved LightGBM model: performance analysis and comparison. IEEE Access. 2023;11:23366–80.

[3] Gudigar A, Nayak S, Samanth J, Raghavendra U, AJA, Barua PD, et al. Recent Trends in Artificial Intelligence-Assisted Coronary Atherosclerotic Plaque Characterization. Int J Environ Res Public Health. 2021;18(19):10003. doi: 10.3390/ijerph181910003 34639303 PMC8508413

[4] Ovalle-Magallanes E, Avina-Cervantes JG, Cruz-Aceves I, Ruiz-Pinales J. Transfer Learning for Stenosis Detection in X-ray Coronary Angiography. Mathematics. 2020;8(9):1510. doi: 10.3390/math8091510

[5] Hossain MB, Iqbal SMHS, Islam MM, Akhtar MN, Sarker IH. Transfer learning with fine-tuned deep CNN ResNet50 model for classifying COVID-19 from chest X-ray images. Inform Med Unlocked. 2022;30:100916. doi: 10.1016/j.imu.2022.100916 35342787 PMC8933872

[6] Apostolopoulos ID, Apostolopoulos DI, Spyridonidis TI, Papathanasiou ND, Panayiotakis GS. Multi-input deep learning approach for Cardiovascular Disease diagnosis using Myocardial Perfusion Imaging and clinical data. Physica Medica. 2021;84:168–77. doi: 10.1016/j.ejmp.2021.04.011 33901861

[7] Algarni M, Al-Rezqi A, Saeed F, Alsaeedi A, Ghabban F. Multi-constraints based deep learning model for automated segmentation and diagnosis of coronary artery disease in X-ray angiographic images. PeerJ Comput Sci. 2022;8:e993. doi: 10.7717/peerj-cs.993 35721418 PMC9202622

[8] Gao Z, Wang L, Soroushmehr R, Wood A, Gryak J, Nallamothu B, et al. Vessel segmentation for X-ray coronary angiography using ensemble methods with deep learning and filter-based features. BMC Med Imaging. 2022;22(1):10. doi: 10.1186/s12880-022-00734-4 35045816 PMC8767756

[9] Danilov VV, Klyshnikov KY, Gerget OM, Kutikhin AG, Ganyukov VI, Frangi AF, et al. Real-time coronary artery stenosis detection based on modern neural networks. Sci Rep. 2021;11(1):7582. doi: 10.1038/s41598-021-87174-2 33828165 PMC8027436

[10] Stralen PV, Rodrigues DL, Oliveira AL, Menezes MN, Pinto FJ. Stenosis Detection in X-ray Coronary Angiography with Deep Neural Networks Leveraged by Attention Mechanisms. In: Proceedings of the 9th International Conference on Bioinformatics Research and Applications, 2022. 123–8. 10.1145/3569192.3569212

[11] Kumar R, Dhiman G. A comparative study of fuzzy optimization through fuzzy number. International Journal of Modern Research. 2021;1(1):1–4.

[12] Shami TM, Grace D, Burr A, Mitchell PD. Single candidate optimizer: a novel optimization algorithm. Evol Intel. 2022;17(2):863–87. doi: 10.1007/s12065-022-00762-7

[13] Zhang Y, Zhou X, Shih P-C. Modified Harris Hawks Optimization Algorithm for Global Optimization Problems. Arab J Sci Eng. 2020;45(12):10949–74. doi: 10.1007/s13369-020-04896-7

[14] Qu C, He W, Peng X, Peng X. Harris Hawks optimization with information exchange. Applied Mathematical Modelling. 2020;84:52–75. doi: 10.1016/j.apm.2020.03.024

[15] Sihwail R, Omar K, Ariffin KAZ, Tubishat M. Improved Harris Hawks Optimization Using Elite Opposition-Based Learning and Novel Search Mechanism for Feature Selection. IEEE Access. 2020;8:121127–45. doi: 10.1109/access.2020.3006473

[16] Fan Q, Chen Z, Xia Z. A novel quasi-reflected Harris hawks optimization algorithm for global optimization problems. Soft Comput. 2020;24(19):14825–43. doi: 10.1007/s00500-020-04834-7

[17] Al-Safi H, Munilla J, Rahebi J. Harris Hawks Optimization (HHO) Algorithm based on Artificial Neural Network for Heart Disease Diagnosis. In: 2021 IEEE International Conference on Mobile Networks and Wireless Communications (ICMNWC), 2021. 1–5. 10.1109/icmnwc52512.2021.9688348

[18] Alsafi H, Alsalihi H, Munilla J. Harris hawks optimization-based ANN for disease diagnosis. In: Proc Int Conf Intell Syst New Appl, 2024. 1–10.

[19] Balamurugan R, Ratheesh S, Venila YM. RETRACTED ARTICLE: Classification of heart disease using adaptive Harris hawk optimization-based clustering algorithm and enhanced deep genetic algorithm. Soft Comput. 2021;26(5):2357–73. doi: 10.1007/s00500-021-06536-0

[20] Kumar AS, Rekha R. An improved hawks optimizer based learning algorithms for cardiovascular disease prediction. Biomedical Signal Processing and Control. 2023;81:104442. doi: 10.1016/j.bspc.2022.104442

[21] Prasanna KSL, Challa NP. Deep Bi-LSTM with Binary Harris Hawkes Algorithm-Based Heart Risk Level Prediction. SN COMPUT SCI. 2023;5(1). doi: 10.1007/s42979-023-02497-3

[22] Maleki S, Zare Mehrjerdi Y. Diagnosis of Coronary Artery Disease by Bat and Harris Hawk Meta-Heuristic Optimization Algorithms and Machine Learning Methods. JHA. 2022;25(1):57–68. doi: 10.52547/jha.25.1.57

[23] Vijayaraj AR, Pasupathi S. Nature inspired optimization in context-aware-based coronary artery disease prediction: A novel hybrid Harris Hawks approach. IEEE Access. 2024;12:92635–51.

[24] Fu W, Zhang K, Wang K, Wen B, Fang P, Zou F. A hybrid approach for multi-step wind speed forecasting based on two-layer decomposition, improved hybrid DE-HHO optimization and KELM. Renewable Energy. 2021;164:211–29. doi: 10.1016/j.renene.2020.09.078

[25] Moayedi H, Osouli A, Nguyen H, Rashid ASA. A novel Harris hawks’ optimization and k-fold cross-validation predicting slope stability. Engineering with Computers. 2019;37(1):369–79. doi: 10.1007/s00366-019-00828-8

[26] Popov M, Amanturdieva A, Zhaksylyk N, Alkanov A, Saniyazbekov A, Aimyshev T, et al. Dataset for Automatic Region-based Coronary Artery Disease Diagnostics Using X-Ray Angiography Images. Sci Data. 2024;11(1):20. doi: 10.1038/s41597-023-02871-z 38172163 PMC10764944

[27] Ovalle-Magallanes E, Avina-Cervantes JG, Cruz-Aceves I, Ruiz-Pinales J. Adv Intell Dis Diagn Treat. Springer. 2024:119–41.

[28] Jiménez‐Partinen A, Molina‐Cabello MA, Thurnhofer‐Hemsi K, Palomo EJ, Rodríguez‐Capitán J, Molina‐Ramos AI, et al. CADICA: A new dataset for coronary artery disease detection by using invasive coronary angiography. Expert Systems. 2024;41(12). doi: 10.1111/exsy.13708

[29] Tao X, Dang H, Zhou X, Xu X, Xiong D. A Lightweight Network for Accurate Coronary Artery Segmentation Using X-Ray Angiograms. Front Public Health. 2022;10:892418. doi: 10.3389/fpubh.2022.892418 35692314 PMC9174536

[30] Sait AR, Awad AM. Ensemble learning-based CAD detection using CT images. Appl Sci. 2024;14(3):1238.

[31] Sharma H, Bharali J, Motghare M, Arora K, Yoon C, Joshi GP. Development and evaluation of hybrid harris hawks optimization algorithms for advanced engineering applications. Sci Rep. 2025;15(1):43123. doi: 10.1038/s41598-025-23624-5 41339382 PMC12678781

[32] Zhang Q, Sheng J, Zhang Q. Improved Harris Hawks optimization for disease detection. Artif Intell Rev. 2025;58:301.

[33] Houssein EH, Mohamed M, Younis EMG, Mohamed WM. A hybrid Harris Hawks Optimization with Support Vector Regression for air quality forecasting. Sci Rep. 2025;15(1):2275. doi: 10.1038/s41598-025-86275-6 39824922 PMC11742066

[34] Dörterler S, Dumlu H, Özdemir D, Temurtaş H. Hybridization of Meta-heuristic Algorithms with K-Means for Clustering Analysis: Case of Medical Datasets. GMBD. 2024;10(1):1–11. doi: 10.30855/gmbd.0705n01

[35] Kowalski PA, Kucharczyk S, Mańdziuk J. Constrained Hybrid Metaheuristic Algorithm for Probabilistic Neural Networks learning. Information Sciences. 2025;713:122185. doi: 10.1016/j.ins.2025.122185

